# Dr. Baron on the Identity of Small-Pox and Cow-Pox

**Published:** 1839-01

**Authors:** John Baron


### PART FOURTH.

### Medical Intelligence.

---

### ON THE IDENTITY OF SMALL-POX AND COW-POX.

By John Baron, M.D. F.R.S. &c.

(*In a Letter addressed to the Editors.*)

Gentlemen, *Cheltenham; Dec.* 4, 1838.

Permit me to offer my best thanks for the kind and respectful manner in which you have been pleased to speak of my Life of Jenner. The spirit you have evinced on this occasion, leaves no room to doubt, that you will candidly give a place to the following remarks in your next Number. They are intended to rectify some mistakes into which the reviewer has inadvertently fallen in discussing the identity of variola and the variolæ vaccinæ.

You will not be surprised when I repeat my conviction that the proofs, both historical and pathological, by which that identity is illustrated, amount very nearly to a complete demonstration. To go through all the links in this chain would on the present occasion be impossible; but as I shall be enabled in a few words to remove the main objections which you offer to the doctrine, I am sure you will not refuse me an opportunity of doing so. Were the question merely of a speculative character I would not trouble you, but as it has a direct bearing upon the lives of our fellow creatures, you will I doubt not hail every opportunity of diffusing truth.

After quoting my opinion respecting the result of the blunders that were committed at the commencement of vaccination in the Small-Pox Hospital, you observe, p. 486, "Two facts are here put in apposition by Dr. Baron; one that the cow-pox sometimes affects the milkers with a severe disease; the other that small-pox after repeated inoculations approximates in its nature to the mildness of cow-pox; the number of pustules gradually diminishing until only one is seen, and that at the place of insertion efficiently protecting the constitution from subsequent small-pox, although running its course without constitutional disturbance. Had the result followed the inoculation of pure small-pox virus, the support thence afforded to Dr. Baron's and Dr. Jenner's views of the identity of the two diseases would have been vastly greater; but in the case referred to it was not true small-pox that was inoculated."

I perceive the grounds on which you make this last assertion, but I can entertain no doubt that they are erroneous: for if you will turn to another part of my book, vol. i. p. 287, you will find that small-pox was propagated by contagion from this very virus, and produced fatal results. But take another example, where all questioning such as you have suggested must vanish. The great object of small-pox inoculation was to reduce the number of pustules, and expert inoculators always endeavour to accomplish this object. That it was accomplished we know for certain; and the early inoculators were quite assured that one correct small-pox pustule was capable of protecting the constitution. On this point, I observe, vol. i. p. 245, "as therefore the variolæ vaccinæ sometimes assumes the character of small-pox under one of its modifications, so the latter under certain circumstances approximates in its nature the mildness of the former. *After a series of inoculations with true variolous matter, it has often been observed that the severity of the symptoms and the number of the pustules gradually diminish till only one is seen at the point of insertion.*"

Again, you say, at p. 495, that the artificial communication of the cow-pox has never produced a general eruption or true small-pox. I perceive from this statement that you have overlooked the information contained in the appendix to my second volume, p. 455, where I say "I have in this volume mentioned the variolous epizootic in Bengal, and the propagation of the genuine variolæ vaccinæ from that source. I have since received more recent intelligence from the same quarter, which proves that more extensive inoculations from the diseased cows have produced not the mild vaccine vesicle but an eruptive disease of the true variolous character. When the black cattle in England were affected in 1780 with a destructive variolous complaint, there can be no doubt that inoculation from this disease would have produced similar results. Dr. Jenner, at a later period, found the variolæ among the cows of a more mild and less malignant nature. He employed this mild virus, and with what success all the world knows."

There is still later intelligence upon the same subject from India. Mr. J. Wood, of Gowalpara, in a paper published in Mr. Corbyn's India Journal for March, details several cases in which lymph taken from the cows produced a disease of an eruptive character. In one of the cases, that of a fine healthy native boy five years of age, Mr. Wood was not without apprehension that it would terminate fatally, from the violence of the incursive fever. In other respects the symptoms closely resemble those of small-pox, *so as to confirm the suspicion of Dr. Jenner, that the small-pox and cow-pox virus might have both at first originated in the same source and be essentially of the same nature.*

From various trials, at different places, Mr. Wood is of opinion that cow-pox is not invariably and uniformly so very safe a prophylactic against small-pox in India as it has been found in Europe, and that, if such instances multiply, it might be a question whether it may not be prudent to resort to small-pox inoculation at times when the cow-pox assumes this dangerous and fatal form. From a few trials, he is inclined to think that at such times, it is preferable to vaccination, inasmuch as it has produced a milder and safer disease. The risk of creating an epidemic small-pox thereby, he thinks too small to merit notice.

The ravages occasioned by small-pox throughout India are frightful. In Ajmeer, during six weeks of the present year, nearly 3,000 deaths occurred through it.* I can scarcely conceive any testimony more striking than that afforded by Mr. Wood; he is manifestly an unprejudiced witness, and the conclusions he has arrived at have been forced upon him by the phenomena which he witnessed, and that too in direct opposition to his previously conceived opinions.

Permit me to draw your attention to another misapprehension which appears in the review, where it is said that the *Grease* is the form in which the disease appears in the horse. I have taken some pains to correct this mistake in the Appendix above referred to, where I remark, "I take this opportunity of expressing my regret that I have employed the word *Grease* in alluding to the disease in the horse. Variolæ equinæ is the proper designation. It has no necessary connexion with the grease though the disorders frequently coexist. This circumstance at first misled Dr. Jenner, and it has caused much misapprehension and confusion." I have moreover shown, vol. i. p. 242, that the variolæ equinæ is a vesicular disease and is sometimes diffused over the body, not being confined to the heels which is the case with the grease.

In conclusion, I have to observe, that as the true small-pox has been conveyed from the cow to man, so in like manner has the disease been communicated from man to the cow. I do not at present refer either to the experiments of Mons. Viborg or Professor Sonderland, but to facts mentioned at p. 441 of my first volume, on the authority of Dr. Waterhouse. At one of the periodical inoculations in New England, cows were milked by persons in all stages of the small-pox: the consequence was, that the cows had an eruptive disorder on their teats and udders so like the small-pox pustule, that every one in the hospital, as well as the physician who told me, declared that the cows had the small-pox.

Several other remarks have occurred to me, on reading other portions of the review, but I hope it is unnecessary to bring them forward at present; I trust what is said

* See Asiatic Journal for December, 1838; p. 970.

will induce my professional brethren to examine all the evidence accumulated in the life of Jenner, deliberately and impartially, and I can entertain no fear as to the result. Truth has been the only object of my search, and if that be obtained, I shall be satisfied.

I am, gentlemen, yours faithfully,

J. BARON.

---

